# Pharmacological corticosterone suppresses pro-inflammatory cytokine gene expression in a tissue-dependent manner independent of sleep fragmentation in male mice

**DOI:** 10.1242/bio.062757

**Published:** 2026-07-28

**Authors:** Hunter J. Weaver, Kimberly A. Turner, Van Thuan Nguyen, Noah T. Ashley

**Affiliations:** Department of Biological Sciences, Western Kentucky University, Bowling Green, KY 42101, USA

**Keywords:** Cytokines, Inflammation, Glucocorticoids, Sleep loss, Sleep fragmentation

## Abstract

Disrupted sleep from obstructive sleep apnea or insomnia promotes an inflammatory environment. Studies in mice have shown that experimental sleep fragmentation (SF) increases pro-inflammatory cytokine gene expression in the brain and peripheral tissues as well as increases circulating glucocorticoids. Although glucocorticoids classically exert anti-inflammatory effects at pharmacological concentrations, they may also modulate immune function in a context- and dose-dependent manner. To investigate whether glucocorticoids regulate pro-inflammatory responses, we used a classical adrenalectomy (ADX) ablation and corticosterone (CORT) replacement design. Adult male C57BL/6J mice received bilateral ADX, sham surgery, or ADX with CORT replacement in drinking water and were exposed to acute (24 h) SF or no SF (NSF). Pro-inflammatory gene expression (interleukin-1 beta and tumor necrosis factor-alpha) was quantified in the hippocampus, hypothalamus, and prefrontal cortex, as well as peripheral tissues including the liver, spleen, heart, and epididymal white adipose tissue. ADX effectively reduced serum CORT, while CORT replacement produced pharmacological hormone levels. Pharmacological CORT treatment robustly suppressed pro-inflammatory gene expression across multiple brain regions (prefrontal cortex, hypothalamus) and peripheral tissues (liver, heart, spleen) compared with ADX and/or sham mice, indicating a strong anti-inflammatory effect at elevated glucocorticoid concentrations. In contrast, ADX mice showed modest, tissue-specific increases in inflammatory gene expression, primarily in the liver and heart, suggesting limited regulation by physiological CORT levels. SF did not significantly increase pro-inflammatory gene expression compared with controls. In conclusion, CORT exerts potent, dose-dependent, and tissue-specific suppression of pro-inflammatory gene expression that is not modulated by acute SF in male mice.

## Teaser

**Summary:** Pharmacological corticosterone suppressed pro-inflammatory cytokines in brain and peripheral tissues, independent of sleep fragmentation, revealing tissue-dependent glucocorticoid immune regulation.

## INTRODUCTION

Sleep is an essential and ubiquitous biological phenomenon, but its precise function is not well understood (Cirelli and Tononi, 2008). When sleep is disrupted or prevented, deleterious effects can accumulate, such as reduced antioxidant concentration (Everson et al., 2005), diminished immune function (Irwin et al., 1996), and reduced cognitive performance (Ellenbogen, 2005). A large body of evidence indicates that chronic sleep dysfunction associated with obstructive sleep apnea (OSA), shift work, and/or light pollution can be harmful (Kecklund and Axelsson, 2016; Khan and Aouad, 2022; Walker et al., 2020), predisposing individuals to the development of cardiovascular and metabolic disease, obesity, and neurological disorders. Even short-term sleep loss can lead to the development of poor behavioral and physiological outcomes (Cedernaes et al., 2018; Craven et al., 2022; Lamon et al., 2021; Liu et al., 2023).

One proposed mechanism linking sleep disruption to disease is altered immune regulation. Sleep loss has been frequently associated with changes in inflammatory signaling in both central and peripheral tissues, although findings are variable across experimental conditions, tissues, and durations of sleep disturbance (Faraut et al., 2012; Garbarino et al., 2021; Hurtado-Alvarado et al., 2013). It has been hypothesized that cytokine signaling follows a circadian pattern, with pro-inflammatory mediators increasing in the active phase and potentially accumulating in the absence of sleep (Besedovsky et al., 2012). This effect has been demonstrated in a wide variety of vertebrates, such as humans, mice, rats, and birds (Cooper et al., 2019; Dumaine and Ashley, 2015; Irwin et al., 2016; Zielinski et al., 2014). However, the extent to which sleep disruption consistently induces a pro-inflammatory phenotype remains unclear.

Sleep fragmentation (SF), restriction, or deprivation can trigger the physiological stress response, which involves the activation of the sympathetic nervous system (SNS) and the hypothalamic-pituitary-adrenal (HPA) axis (Meerlo et al., 2008). While prior work from our laboratory has implicated the SNS in mediating inflammatory responses to sleep disruption (Ensminger et al., 2022; Mishra et al., 2022, 2020), the contribution of glucocorticoid signaling remains less well defined. Glucocorticoids, released from the adrenal cortex following HPA axis activation, are classically considered immunosuppressive; however, their effects are highly context and dose dependent (Cain and Cidlowski, 2017).

Since the HPA axis is rapidly activated during certain forms of sleep loss, it is possible that glucocorticoids could modulate, rather than uniformly suppress, inflammatory responses from sleep loss. Consistent with this complexity, pharmacological manipulation of glucocorticoid signaling produced tissue-dependent inflammatory effects in sleep-fragmented mice (Hasan et al., 2024), suggesting a nonuniform regulatory role that glucocorticoid levels play across organ systems. However, whether endogenous versus pharmacological glucocorticoid levels differentially regulate inflammatory responses to SF remains unclear.

The current study aimed to investigate the effect of glucocorticoid signaling in regulating pro-inflammatory responses to acute SF using a classical adrenalectomy (ADX) and corticosterone (CORT) replacement approach. Adult male C57BL/6J mice underwent bilateral ADX, sham surgery (sham), or ADX with exogenous CORT in drinking water (ADX+CORT) and were exposed to either acute SF or control conditions [no SF (NSF)]. Pro-inflammatory cytokine gene expression [interleukin-1 beta (IL-1β) and tumor necrosis factor-alpha (TNF-α)] was assessed in the hippocampus, hypothalamus, and prefrontal cortex (PFC), as well as peripheral tissues including the liver, spleen, heart, and epididymal white adipose tissue (EWAT). We hypothesized that if glucocorticoids suppress SF-induced inflammatory responses, then ADX mice would exhibit increased cytokine gene expression relative to sham controls, with CORT replacement reversing this effect. Alternatively, if glucocorticoids potentiate inflammatory signaling under conditions of SF, then opposing effects could be expected. We further assessed whether acute SF alters inflammatory gene expression across multiple tissues. Taken together, this design allows dissociation of endogenous versus pharmacological effects on tissue-specific inflammatory responses to acute SF.

## RESULTS

### Serum CORT concentration

**Fig. 1.**
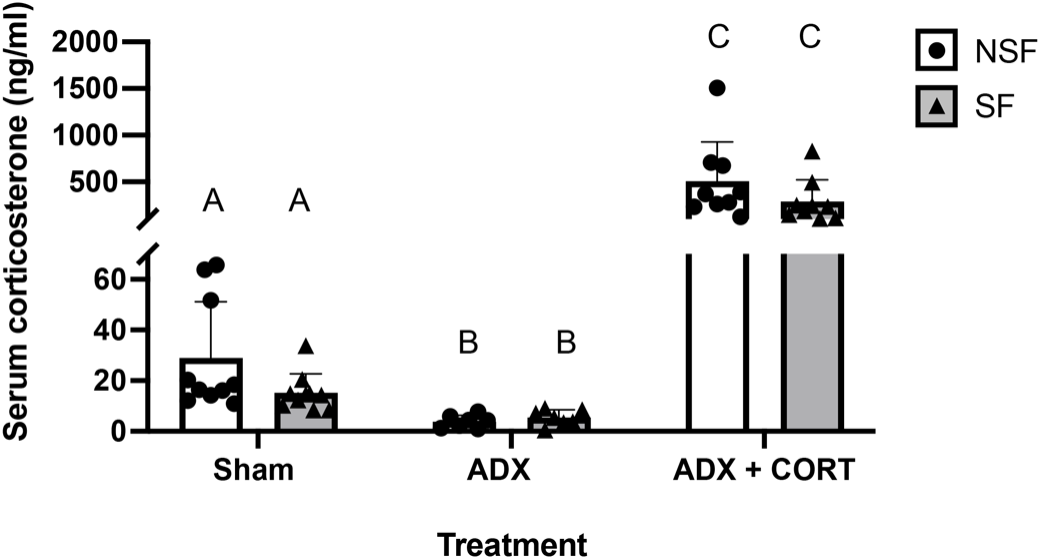
**Serum CORT concentration.** Effects of acute SF (SF or NSF) and CORT treatment (sham, ADX or ADX+CORT) on serum CORT levels. Samples were analyzed using a two-way ANOVA and Tukey's post hoc tests after logarithmic transformation. Data are back-transformed and shown as ±1 s.e. for each group, and differing letters denote *P*<0.05.

There was a significant effect of CORT treatment on serum CORT levels (*F*_2,42_=162.4, *P*<0.0001), but no effects from sleep treatment (*F*_1,42_=1.40, *P*=0.24) or the interaction between main effects (*F*_2,42_=1.66, *P*=0.20; Fig. 1). Post hoc tests showed that ADX mice exhibited significantly lower levels [NSF (mean±s.e.m.), 3.83±2.48 ng/ml; SF, 5.49±3.07 ng/ml] than sham (NSF, 28.96±22.15 ng/ml; SF, 15.25±7.46 ng/ml) or ADX+CORT (NSF, 505.67±422.85 ng/ml; SF, 290.42±232.72 ng/ml; Tukey’s post hoc tests, all *P*<0.0003; Fig. 1). Sham groups were also significantly lower than ADX+CORT groups (Tukey tests, all *P*<0.0001; Fig. 1).

### Brain responses

#### PFC

**Fig. 2.**
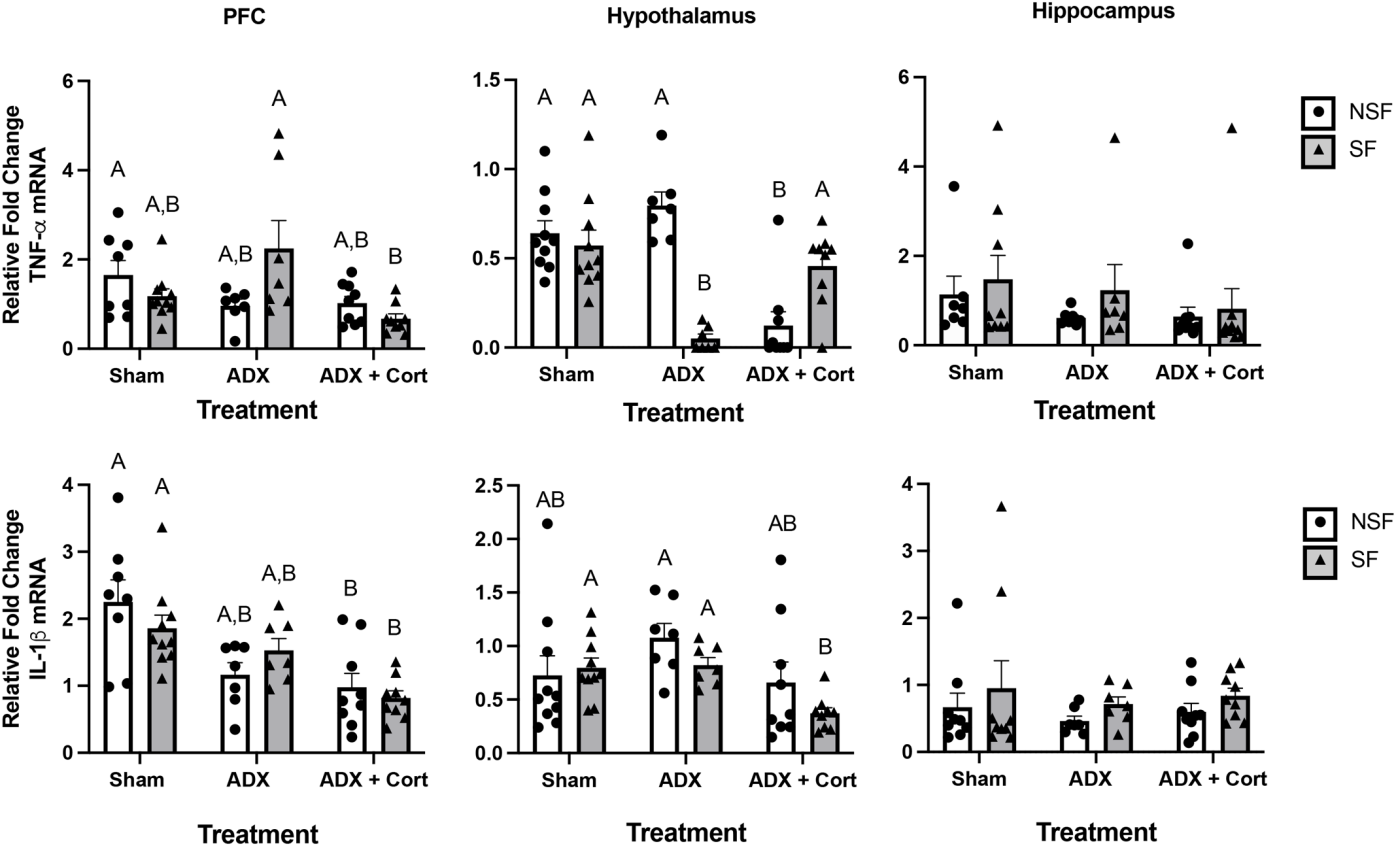
**Brain mRNA expression.** Effects of acute SF (SF or NSF) and CORT treatment (sham, ADX or ADX+CORT) on TNF-α and IL-1β mRNA expression in the PFC, hypothalamus, and hippocampus of male mice. Samples were analyzed using a two-way ANOVA and Tukey's post hoc tests after logarithmic transformation. Data are back-transformed and shown as ±1 s.e. for each group, and differing letters denote *P*<0.05.

**Fig. 3.**
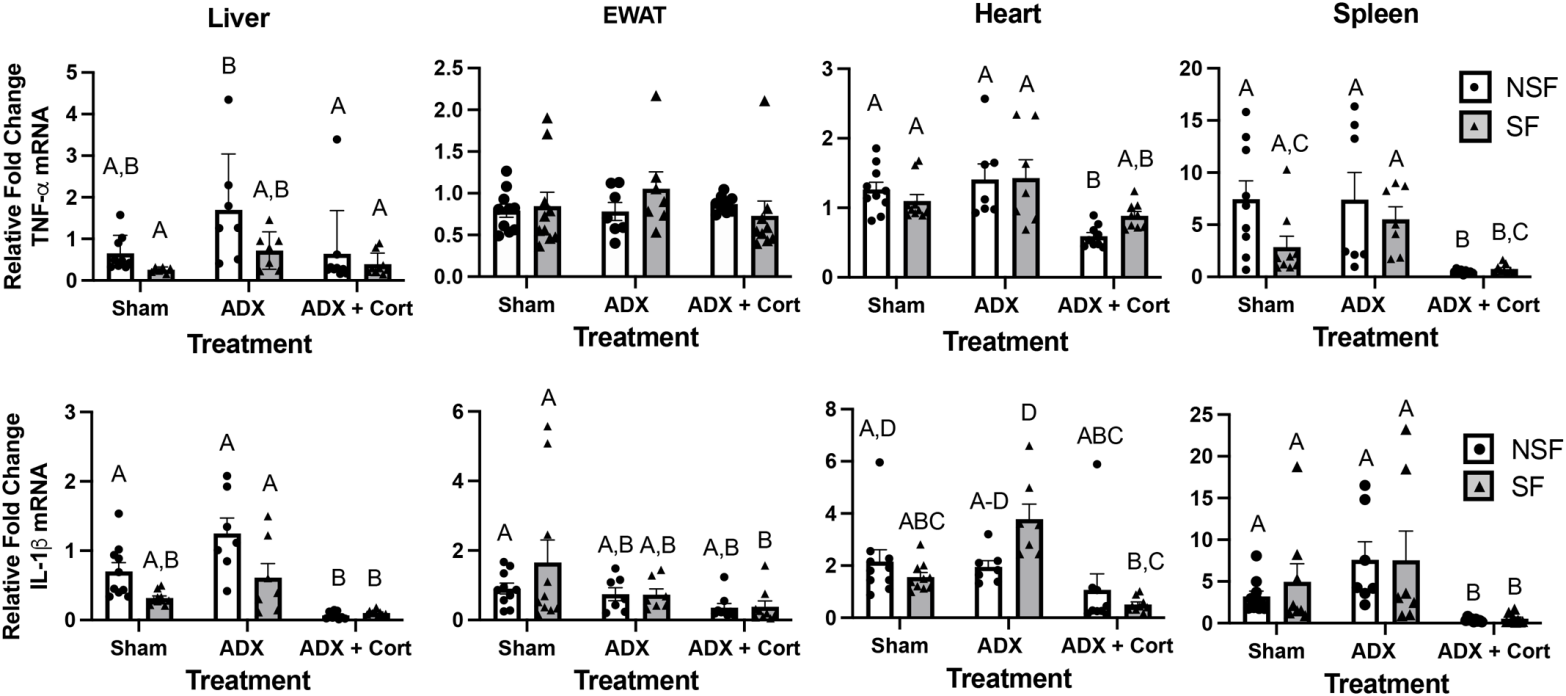
**Peripheral mRNA expression.** Effects of acute SF (SF or NSF) and CORT treatment (sham, ADX or ADX+CORT) on TNF-α and IL-1β mRNA expression levels in the liver, EWAT, heart, and spleen. Samples were analyzed using a two-way ANOVA and Tukey's post hoc tests after logarithmic transformation. Data are back-transformed and shown as ±1 s.e. for each group, and differing letters denote *P*<0.05.

SF had no effect on TNF-α and IL-1β gene expression levels (*F*_1,44_=0.04, *P*=0.85, and *F*_1,44_=0.12, *P*=0.73, respectively, Fig. 3), but CORT treatment significantly affected the gene expression of both cytokines (*F*_2,44_=4.59, *P*=0.01 and *F*_2,44_=15.78, *P*<0.0001, respectively, Fig. 2). There was also a significant interaction effect between sleep and CORT treatments upon TNF-α expression (*F*_2,44_=5.20, *P*=0.0009). ADX+CORT mice exposed to SF had significantly reduced TNF-α expression compared with ADX SF mice (Tukey's post hoc test, *P*=0.004) and sham NSF mice (*P*=0.04). IL-1β gene expression was also significantly lower in ADX+CORT mice compared with that of sham groups (Tukey's post hoc, all *P*<0.005) but not that of ADX mice (all *P*>0.09).

#### Hypothalamus

Hypothalamic TNF-α expression was significantly affected by CORT treatment (*F*_2,46_=7.32, *P*=0.002) but not by SF (*F*_1,46_=0.00, *P*=0.99; Fig. 2). A significant interaction between main effects was also observed (*F*_2,46_=13.82, *P*<0.0001). Post hoc analyses indicated that among SF mice, ADX+CORT enhanced TNF-α expression compared with ADX mice, suggesting a pro-inflammatory effect of CORT (Tukey's post hoc test, *P*=0.046; Fig. 2). However, among NSF mice, the opposite effect occurred, whereby ADX+CORT treatment suppressed TNF-α expression compared with ADX mice, suggesting an anti-inflammatory effect (Tukey's post hoc test, *P*=0.0001; Fig. 2).

Sleep treatment had no effect on hypothalamic IL-1β gene expression (*F*_1,46_=0.47, *P*=0.50; Fig. 2), and CORT treatment showed a significant effect (*F*_2,46_=8.98, *P*=0.0005), but no interaction effect was observed (*F*_2,46_=1.58, *P*=0.22; Fig. 2). ADX+CORT mice exposed to SF displayed reduced IL-1β gene expression levels compared with ADX mice (Tukey's post hoc test, all *P*<0.04) and sham SF mice (*P*=0.04).

#### Hippocampus

There were no significant effects of sleep treatment, CORT treatment or their interaction on hippocampal TNF-α and IL-1β expression (TNF-α: *F*_1,43_=0.12, *P*=0.74, *F*_2,43_=3.05, *P*=0.06 and *F*_2,43_=0.36, *P*=0.70; IL-1β: *F*_1,44_=2.72, *P*=0.11, *F*_2,44_=0.29, *P*=0.75 and *F*_2,44_=0.42, *P*=0.66, respectively; Fig. 2).

### Peripheral responses

#### Liver

Sleep treatment and CORT treatment had a significant effect on hepatic TNF-α gene expression (*F*_1,41_=7.55, *P*=0.009, *F*_2,41_=7.99, *P*=0.001, respectively), but there was no significant interaction effect (*F*_2,41_=1.39, *P*=0.27; Fig. 3). ADX NSF mice showed increased TNF-α expression compared with sham SF and ADX+CORT mice (Tukey's post hoc, all *P*<0.02; Fig. 3).

CORT treatment (*F*_2,46_=61.02, *P*<0.0001), but not sleep treatment (*F*_1,46_=3.62, *P*=0.06), had a significant effect on hepatic IL-1β gene expression (Fig. 3). The interaction between main factors was also significant (*F*_2,46_=8.18, *P*=0.0009). ADX+CORT groups displayed significantly reduced hepatic IL-1β gene expression compared with ADX groups and sham NSF mice (Tukey's post hoc tests, all *P*<0.002).

#### EWAT

Sleep treatment, CORT treatment, and their interaction had no significant effects upon EWAT TNF-α gene expression (*F*_1,46_=0.06, *P*=0.80, *F*_2,46_=0.47, *P*=0.62, *F*_2,46_=1.99, *P*=0.15, respectively; Fig. 3). EWAT IL-1β gene expression was significantly affected by CORT treatment (*F*_2,46_=8.68, *P*=0.006), but not sleep treatment (*F*_1,46_=0.02, *P*=0.9) or the interaction effect (*F*_2,46_=0.17, *P*=0.85; Fig. 3). ADX+CORT mice receiving SF exhibited reduced EWAT IL-1β gene expression compared with sham groups (Tukey's post hoc test, all *P*<0.045; Fig. 3).

#### Heart

CORT, but not sleep treatment, had an effect upon cardiac TNF-α and IL-1β gene expression (TNF-α: *F*_2,46_=19.07, *P*<0.0001, *F*_1,46_=1.00, *P*=0.32, respectively; IL-1β: *F*_2,46_=11.97, *P*<0.0001, *F*_1,46_=0.46, *P*=0.50, respectively; Fig. 3). There was a significant interaction effect upon cardiac TNF-α and IL-1β gene expression (*F*_2,46_=2.43, *P*=0.02, *F*_2,46_=5.17, *P*=0.01, respectively). ADX+CORT mice experiencing NSF exhibited significantly lower cardiac TNF-α gene expression than sham and ADX groups (Tukey's post hoc tests, all *P*<0.0008; Fig. 3). ADX mice exposed to SF exhibited higher cardiac IL-1β gene expression than ADX+CORT groups and sham SF mice (Tukey's post hoc test, all *P*<0.007; Fig. 3). Sham mice exposed to NSF exhibited higher cardiac IL-1β gene expression than ADX+CORT mice exposed to SF (Tukey's post hoc test, *P*=0.048).

#### Spleen

Sleep treatment and the interaction between sleep and CORT treatments had no significant effect upon splenic TNF-α or IL-1β gene expression (TNF-α: *F*_1,44_=0.75, *P*=0.39, *F*_2,44_=3.11, *P*=0.054; IL-1β: *F*_1,44_=0.24, *P*=0.63, *F*_2,44_=0.42, *P*=0.66, respectively), but CORT treatment significantly affected expression of both genes (*F*_2,44_=31.35, *P*<0.0001, *F*_2,44_=43.32, *P*<0.0001, respectively; Fig. 3). Splenic TNF-α expression was reduced in ADX+CORT mice compared with sham NSF mice and ADX mice (Tukey's post hoc test, all *P*<0.006). ADX+CORT mice exposed to NSF also displayed reduced splenic TNF-α expression compared with sham SF mice (Tukey's post hoc test, *P*=0.008). Splenic IL-1β gene expression was suppressed in ADX+CORT groups compared with sham and ADX groups (Tukey's post hoc test, all *P*<0.0001).

## DISCUSSION

The results of this study indicate that glucocorticoid signaling is a primary regulator of inflammatory gene expression across brain and peripheral tissues in mice exposed to acute SF, with effects that are strongly dependent on both dose and tissue type (Table 1). Pharmacological CORT replacement produced widespread effects of pro-inflammatory cytokine expression in multiple brain regions (PFC and hypothalamus) and peripheral tissues (liver, spleen, heart). In contrast, physiological glucocorticoid signaling appeared to exert more limited control on inflammatory gene expression, with modest and tissue-restricted effects observed primarily in the liver and heart.

**Table 1.** Summary of results indicating significant main effects and interactions as well as comparisons between high (ADX+CORT), medium (sham), and low (ADX) CORT groups and whether there was suppression in cytokine gene expression

		Significant factors			CORT comparison		
		Sleep	CORT	Interaction	High versus low	High versus medium	Medium versus low
Brain							
Prefrontal cortex	TNF-α	No	Yes	Yes	↓	--	--
	IL-1β	No	Yes	No	--	↓	--
Hypothalamus	TNF-α	No	Yes	Yes	↓ ↑*	--	--
	IL-1β	No	Yes	No	↓	↓	--
Hippocampus	TNF-α	No	No	No	--	--	--
	IL-1β	No	No	No	--	--	--
Periphery							
Liver	TNF-α	Yes	Yes	No	↓	--	↓
	IL-1β	No	Yes	Yes	↓	↓	--
EWAT	TNF-α	No	No	No	--	--	--
	IL-1β	No	Yes	No	--	↓	--
Heart	TNF-α	No	Yes	Yes	↓	↓	--
	IL-1β	No	Yes	Yes	↓	↓	↓
Spleen	TNF-α	No	Yes	No	↓	↓	--
	IL-1β	No	Yes	No	↓	↓	

A downward arrow indicates suppression relative to the other CORT group (even if there are differences in how NSF versus SF groups responded). An upward arrow indicates an enhancement effect in cytokine gene expression (relative to the other CORT group).

*In the hypothalamus, SF mice exhibited pro-inflammatory (upward arrow) effects from CORT treatment, whereas NSF mice exhibited anti-inflammatory (downward arrow) effects.

Across brain regions, glucocorticoid effects were heterogeneous. The PFC showed consistent suppression of pro-inflammatory cytokine expression, whereas the hypothalamus exhibited a more complex response profile, and the hippocampus was largely unresponsive. These findings suggest region-specific sensitivity to glucocorticoids, potentially reflecting differences in glucocorticoid receptor density, local steroid metabolism, and/or modulation of distinct neuroimmune signaling pathways.

A notable exception to the overall suppressive pattern was observed for hypothalamic TNF-α expression, where CORT increased expression under SF conditions but reduced expression under control conditions (Table 1). This pattern underscores the context-dependent nature of glucocorticoid signaling within the hypothalamus, a brain region that integrates endocrine, autocrine, and immune functions. Such bidirectional interactions are consistent with prior evidence that glucocorticoids can exert biphasic, dose- and state-dependent effects on inflammatory pathways (Munck and Náray-Fejes-Tóth, 1992; Sapolsky et al., 2000). Under these conditions, CORT may interact with ongoing stress-induced signaling cascades to enhance TNF-α expression in specific hypothalamic cell populations, whereas in unstressed animals, it may exert its more typical anti-inflammatory effects.

In contrast to prior studies from our laboratory using similar SF methodologies, acute SF alone did not significantly increase pro-inflammatory gene expression in any tissue examined. This suggests that, under the present experimental conditions, glucocorticoid availability is a stronger determinant of cytokine expression than acute SF per se. Differences from previous findings may reflect methodological variables, such as surgical manipulation, baseline stress, or differences in HPA axis activation between experimental cohorts. The inclusion of a nonsurgical control would help address this potential confound. In addition, it is possible that ADX and ADX+CORT mice respond differently to SF than sham mice, and the different sleep patterns could alter inflammatory pathways. CORT administration has been shown to increase wakefulness and suppress non-rapid eye movement (NREM) sleep in C57BL/6J mice (Yao et al., 2023).

Serum CORT concentration was significantly different between sham and ADX mice, but glucocorticoid concentration was largely detectable in ADX mice. These findings are consistent with reports of extra-adrenal sources of glucocorticoids (e.g. primary lymphoid organs, intestine, skin, brain; Taves et al., 2011). Pharmacological CORT replacement produced marked elevations in circulating hormone levels and clear anti-inflammatory effects, confirming that glucocorticoid availability remains a primary determinant of cytokine expression.

An additional consideration is that multiple biological endpoints were assessed across several tissues, increasing the total number of statistical tests performed and the potential for Type I error. Although appropriate corrections were applied within analyses, these findings should be interpreted in the context of the overall pattern of effects across related markers and tissues. Similarly, the absence of detectable SF×CORT interactions should be interpreted cautiously, as modest interaction effects cannot be excluded.

A limitation of the study is that animals were treated as independent experimental units without explicit incorporation of cage or chamber clustering due to incomplete recording of housing assignments. While this approach is common in similar experiments, it may contribute to the variability observed in the study.

### Conclusions

Collectively, these findings indicate that glucocorticoid signaling exerts potent, dose-dependent, and tissue-specific control over pro-inflammatory gene expression in brain and peripheral tissues, whereas acute SF does not independently drive robust inflammatory activation under the present experimental conditions. Pharmacological glucocorticoid exposure produces broad immunosuppressive effects, while physiological effects provide more limited, tissue-restricted regulation. These results highlight the importance of hormonal status in shaping neuroimmune responses and suggest that glucocorticoid signaling is a dominant regulator of inflammation compared with acute SF in this model. Given well-known sex differences in immune and glucocorticoid signaling (Klein and Flanagan, 2016), future studies should determine whether these regulatory patterns differ in female mice. In addition, studies employing chronic SF models will be important to better recapitulate sustained sleep disruption associated with OSA and to determine how prolonged sleep loss interacts with endocrine regulation over time.

## MATERIALS AND METHODS

### Animals

Male (M) C57BL/6J mice (*n*=60) were used for this study and housed in our colony room (12:12-h light-dark cycle, lights on at 0800, 21°C±1°C) at Western Kentucky University. Mice were weaned at 21 days of age and housed in polypropylene cages with same-sex littermates. Corncob bedding was provided, and the mice were given *ad libitum* food (RM1800, Cincinnati Labs) and normal drinking water. This research project was conducted with the approval of the Institutional Animal Care and Use Committee at Western Kentucky University (#19-14), and procedures followed the National Institutes of Health's ‘Guide for the Use and Care of Laboratory Animals’ and the ARRIVE guidelines.

### Surgical procedure

Adult mice (8-12 weeks of age) were subjected to bilateral adrenalectomies in which each mouse was deeply anesthetized with isoflurane (5% induction) in a closed container followed by clipping of fur away from the surgical site on both sides of the mouse near the last rib and abdominal region. Eye ointment was provided to prevent ocular desiccation. Each mouse was placed in lateral recumbency, followed by a topical alcohol and betadine scrub to prevent infection at the surgical site. Isoflurane flow was adjusted to 2% maintenance levels. Then, a 1-2 mm incision below the last rib was made on the skin and the muscular wall to expose the adrenal gland. After the adrenal gland was removed by gently teasing the organ away from the surrounding fat, the muscular wall was closed with an absorbable suture. Following closure of the muscular wall, wound clips were used to close the skin incisions. Each mouse promptly received 0.5% bupivacaine at the surgical site to aid in pain relief. After one side was completed, the procedure was performed on the contralateral side in an identical manner. After pain medications were administered on the second side, mice received 1.0 ml i.p. injections of Lactated Ringer's to aid in fluid replacement. After receiving fluids, mice were placed in automated SF chambers (Lafayette Instrument Company; Lafayette, IN, USA; model 80390) on heating pads until fully recovered. Buprenorphine was given post-surgery as needed. ADX mice required 0.9% saline for drinking water. A sham ADX group underwent the same surgical procedure, except that the adrenal glands were left intact (provided with normal tap water). A third group received ADX as well as CORT (25 μg/ml; dissolved in 0.9% saline drinking water; Sigma, St. Louis, MO, USA). Mice were assigned randomly to the different surgical treatments. Animals were excluded only according to predefined criteria, including surgical complications, inadequate postoperative recovery, or technical issues that prevented collection or analysis of biological samples. Three mice in each ADX group and one mouse in each ADX+CORT group were removed from the study due to these criteria.

### Acute SF protocol

After a 1-week recovery from surgery, mice were acclimated to SF chambers. Each cage contained no more than five mice with corncob bedding, food, and their respective water treatments *ad libitum*. After acclimation to the SF chambers, mice were subjected to SF (see below; 24 h) or NSF with sample collection the following day at 08:00.

**Fig. 4.**
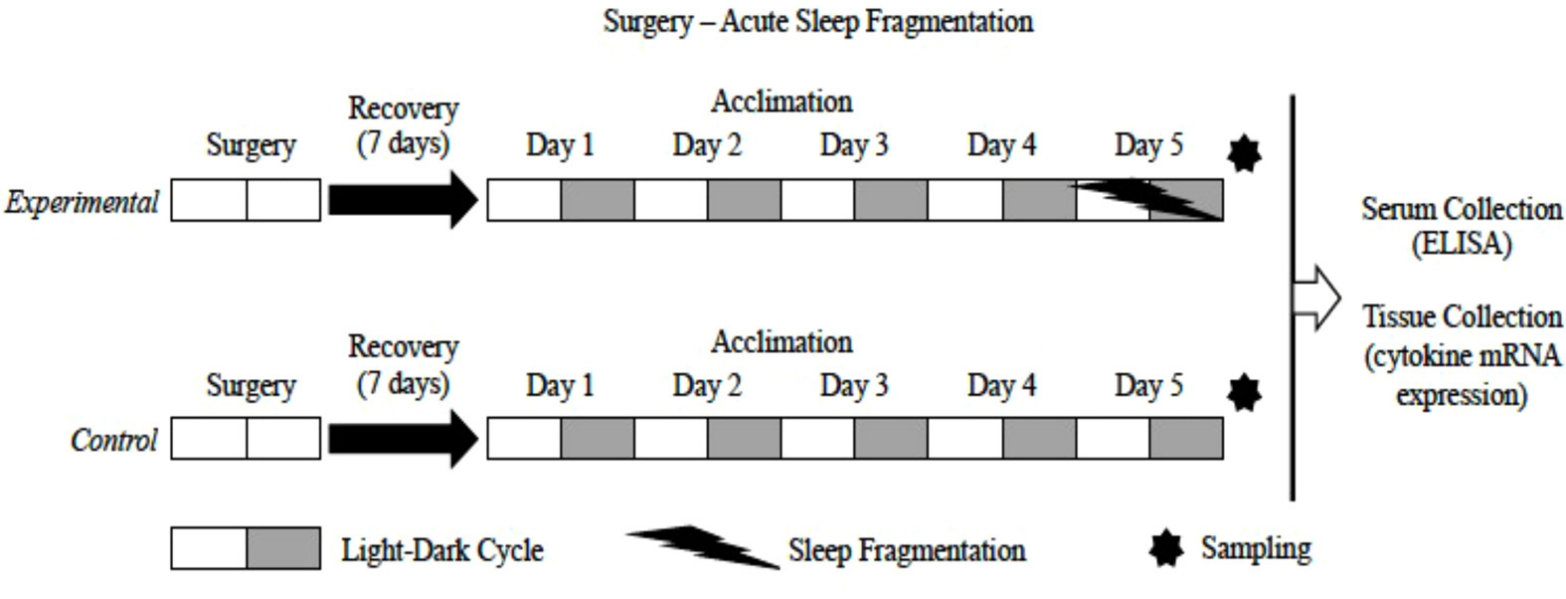
**Experimental protocol for surgery, recovery, and SF experiment.** Before SF was conducted, mice were subjected to bilateral ADX, in which both adrenal glands were removed for ADX and ADX+CORT groups. After 24 h of SF or NSF (control) treatment, mice were euthanized for trunk blood collection and tissue collection.

After acclimation, 26 mice (*n*=10 sham, *n*=7 ADX, and *n*=9 ADX+CORT) were subjected to SF in which the bar sweeps horizontally across the floor of the cage every 120 s (Fig. 4). This rate mimics the degree of SF observed in patients diagnosed with severe OSA (Ramesh et al., 2009; Goyal and Johnson, 2017). Mice typically sleep in between bar sweeps, thus experiencing SF, but not complete sleep deprivation (Mishra et al., 2020). NSF mice (*n*=26; *n*=10 sham, *n*=7 ADX, and *n*=9 ADX+CORT) were not subjected to any bar sweeps but were still housed in the SF chamber.

### Sample collection

After SF or NSF, mice were anesthetized with isoflurane induction (5%) for <1 min and rapidly decapitated in <3 min for tissue gene expression studies and blood collection to measure baseline CORT concentration. Trunk blood was collected from mice kept on ice for less than 20 min and spun at 3000×***g*** for 30 min at 4°C. Serum was collected and stored at −80°C for CORT ELISA analyses (see below). Sample collection and processing order were counterbalanced across groups. For gene expression analyses, three brain regions (PFC, hypothalamus and hippocampus) and four peripheral tissues (liver, EWAT, heart, and spleen) were macrodissected from mice and immediately stored in RNAlater solution (Thermo Fisher Scientific) and then stored at −20°C (within 60 min). In addition, these tissues were coded with a unique numerical identifier that blinded all authors to the experimental groups.

### CORT ELISA

Serum levels of CORT (*n*=7-10/group) were measured as per the manufacturer's protocols (catalog number: ADI-901-097, Enzo Life Sciences). Average intra- and inter-assay coefficients of variation were 6.80% and 5.56%, respectively.

### RNA extraction and gene expression analysis

RNA was extracted from the liver, EWAT, spleen, PFCs, hypothalami and hippocampi using RNeasy mini kits (Qiagen). RNA was isolated from the heart using a fibrous tissue mini kit (Qiagen). Total RNA was reverse transcribed into complementary DNA (cDNA) using a high-capacity cDNA reverse transcription kit (Thermo Fisher Scientific, catalog number: 4368813). The prepared cDNA was used as a template to determine relative cytokine gene expression levels using an ABI 7300 reverse transcriptase PCR (RT-PCR) system. Cytokine probes (IL-1β: Mm00434228_m1, TNF-α: Mm00443258_m1; Thermo Fisher Scientific) labeled with the fluorescent marker 6-carboxyfluorescein (6-FAM) at the 5′ end and a minor groove binder (MGB) quencher at the 3′ end were used for the genes of interest, along with 18S (primer-limited, VIC-labeled probe) as the endogenous control, according to the manufacturer's instructions. Samples were run in duplicate, and the cycle threshold (C_t_) obtained by fluorescence exceeding background levels was used to calculate the relative mRNA expression levels of genes of interest (IL-1β, TNF-α) relative to the endogenous control (18S). The change in mRNA expression levels was determined by ΔΔC_t_ analysis. Outliers were removed if data points were outside ±3 s.d. for the mean of each group.

### Statistical analysis

All statistical analyses were performed using GraphPad Prism (version 9.0). A two-way ANOVA assessed the effect of acute SF (SF versus NSF), the effect of CORT manipulation (sham versus ADX versus ADX+CORT), and their interactions upon CORT concentration and cytokine gene expression. Data were log-transformed to adhere to the principles of normality and homogeneity of variances. Tukey's test was used for post hoc analysis. All animals were treated as independent experimental units, as individual cage assignments were not consistently recorded to assess cage- or chamber-level effects. Results were presented as means±1 s.e., and *P*<0.05 was considered statistically significant.

## Supplementary Material


