# Supplementary material for: Pharmacological corticosterone suppresses pro-inflammatory cytokine gene expression in a tissue-dependent manner independent of sleep fragmentation in male mice
Source: Biol Open. 2026 Jul 28;15(7):bio062757. doi: 10.1242/bio.062757 (PMC13474571; doi:10.1242/bio.062757)
Supplement: Supplementary file 1 [file biolopen-15-062757_review_history.pdf]

Pharmacological corticosterone suppresses pro-inflammatory cytokine gene expression in a tissue-dependent manner independent of sleep fragmentation in male mice

Hunter Weaver, Kimberly A. Turner, Van Thuan Nguyen and Noah Ashley
10.1242/bio.062757

Editor: Kendra J. Greenlee

Review timeline
Original submission: 29 May 2026
Editorial decision: 4 June 2026
Resubmission: 16 June 2026
Accepted: 22 June 2026

Original submission

First decision letter

MS ID#: bio.62608

MS Title: Glucocorticoid mediation of pro-inflammatory responses to sleep fragmentation in mice

Authors: Hunter Weaver; Kimberly Turner; Van Thuan Nguyen; Noah Ashley

I am writing to let you know that I have now reached a decision on the above manuscript. I am afraid that, after careful consideration, I feel that it cannot currently be accepted for publication in Biology Open.

The reviewer reports are shown at the bottom of this email.

As you will see, the reviewers raise a number of substantial criticisms that prevent me from accepting your paper for publication. In particular, both reviewers pointed out that in this study, sleep fragmentation did not induce the pro-inflammatory phenotype, which makes it difficult to test the effects of the glucocorticoid manipulation. In addition, there was no clear reason why the treatment did not work in this study, as this phenotype was routinely observed in previous studies. Concerns with the statistical analysis and methods were also noted.

I realise that this is disappointing news, and we understand the frustration that you must feel. I do hope you find the reviewer comments helpful in allowing you to revise the manuscript for successful submission to Biology Open or elsewhere.

Reviewer 1

Comments for the author

In this manuscript, Weaver et al. use a bilateral adrenalectomy/corticosterone-replacement design to ask whether glucocorticoids regulate pro-inflammatory cytokine gene expression (Il1b, Tnf) in brain and peripheral tissues of male mice exposed to acute sleep fragmentation. The principal findings are that pharmacologically elevated corticosterone broadly suppresses cytokine expression,

© 2026. Published by The Company of Biologists under the terms of the Creative Commons Attribution License (https://creativecommons.org/licenses/by/4.0/).

that differences between sham and adrenalectomized animals are confined to a few tissues, and that sleep fragmentation itself produced essentially no inflammatory effect. The study is technically competent, the analyses are appropriate, and I particularly commend the authors for reporting the null sleep-fragmentation result openly. My substantive comments concern the framing of the work rather than its conduct, and I would support publication after these revisions.

Major
1. The manuscript is framed throughout as a test of glucocorticoid regulation of the inflammatory response to sleep fragmentation, but sleep fragmentation produced little or no such response in this cohort. The experiment therefore does not, in practice, address that question. It does, however, clearly demonstrate that elevated corticosterone suppresses cytokine gene expression across most tissues. I would encourage the authors to foreground this result and to revise the title and the concluding sentence of the Abstract so that the central claim concerns the anti-inflammatory action of corticosterone, with sleep fragmentation as the experimental context rather than the response being modulated. This requires no new data.
2. The replacement protocol produced circulating corticosterone markedly above the intact physiological range, so the strong suppressive effects are best described as pharmacological rather than as a physiological replacement. The authors note this in the Discussion; I ask that the Abstract and Conclusions reflect it consistently, and that the sham-versus-ADX contrast be presented explicitly as the study's window onto physiological glucocorticoid action, an effect detectable in only a small number of tissues.
3. All animals, including the sham group, underwent surgery with approximately one week of recovery. Residual surgical and HPA activation is a plausible explanation for the absent sleep-fragmentation effect, given that the authors have reported such effects previously. A brief acknowledgement of the lack of a non-surgical control would strengthen the Discussion.
4. The hypothalamic TNF-α interaction, in which corticosterone exerted opposing effects under sleep-fragmentation and control conditions, is the most novel observation in the study and currently receives limited treatment. I suggest a short paragraph on biphasic, state-dependent glucocorticoid action, and a clarification of the corresponding entry in Table 1, where the dual arrows are difficult to interpret as presented.
Minor
5. Two reported F-statistics have error degrees of freedom inconsistent with the other effects in the same ANOVA and appear to be typographical errors. Please reconcile.
6. Abstract: "glucocorticoids… has a suppressive effect" should read have.
7. Typographical corrections: "respectfully" → respectively; "son serum" → on (Figure 2 legend); "supressed" → suppressed; and "stratum" → presumably striatum.

Requires the narrative to be aligned with the results. With the framing adjustments above I would be happy to see it published.

<u>Reviewer 2</u>

Comments for the author

The experimental question is important and the adrenalectomy/replacement design is appropriate in principle for testing glucocorticoid involvement. However, this study suffers from several important limitations that preclude publication. First, it does not address the central question as it is currently framed. The major problem is that sleep fragmentation did not reliably induce the expected pro-inflammatory phenotype. This substantially weakens the claim that the study identifies glucocorticoid mediation of inflammatory responses to sleep fragmentation. A more conservative interpretation would be that pharmacological corticosterone replacement suppresses cytokine transcript expression in a tissue-dependent manner.

There are several weaknesses in the design and reporting. First, only male mice were used, which limits inference of the study: sleep, HPA-axis biology and immune signalling, all show sex-dependent effects. The corticosterone replacement group appears to have reached pharmacological rather than physiological levels, making inference of normal glucocorticoid regulation impossible. Incidentally, the study would benefit from clearer evidence that the sleep-fragmentation protocol produced equivalent disruption across groups, especially given that

© 2026. Published by The Company of Biologists under the terms of the Creative Commons Attribution License (https://creativecommons.org/licenses/by/4.0/).

adrenalectomy and corticosterone replacement could themselves alter sleep, arousal or stress responsiveness.

The statistical analysis is not fully convincing as reported. The study includes multiple tissues, two cytokine transcripts, corticosterone measurements and numerous post hoc contrasts, but there does not appear to be a clear correction or control strategy for multiple testing across the full family of outcomes. The manuscript should also clarify whether cage or sleep-fragmentation chamber was treated as a potential experimental unit or blocking factor, since animals were housed in groups and the manipulation may not be fully independent at the individual-animal level. Similarly, the biological replication is a concern. Final group sizes were small and uneven, particularly in the adrenalectomy groups, and no a priori power analysis or effect-size justification is provided. This is especially problematic for detecting sleep × corticosterone interactions. The absence of sleep-fragmentation effects may therefore reflect inadequate power, surgical recovery effects, or both. At the very least it would be expected that there was a power justification, clarity over exclusion criteria and timing, and ideally show individual-level data for all endpoints.

The methods have several important details missing, or a lesat, incompletely reported. The authors state that tissue samples were coded so that gene-expression analyses were blinded, but did they blind all of the allocation to experimental condition, surgery, sample collection, tissue dissection, exclusion decisions and statistical analysis? or how were these randomised? It should also report whether sample collection and processing order were counterbalanced across groups. This is critical detail because corticosterone and inflammatory transcripts are strongly time-sensitive, and although it states that collection was performed at 0800, a single sampling time and unclear collection order limit interpretation.

The conclusions are much too broad. The data support tissue-dependent suppression of cytokine transcript expression by high corticosterone replacement more strongly than they support glucocorticoid mediation of sleep-fragmentation-induced inflammation. The title overstates the result, since the manuscript reports little evidence that sleep fragmentation itself increased pro-inflammatory responses. The abstract is ok, but the title should reflect the actual findings.

Reviewer's Responses to Questions

Experimental quality
Are experiments performed using appropriate methods that will answer the question (or test the hypothesis or support the observations) posed by the authors? Is the right tool used for the job?
If 'No', please indicate reasons in Comments for Author box below.
Reviewer #1:

- Yes

Reviewer #2:

- No

Were the data analyzed using appropriate statistical tests?
If 'No', please indicate reasons in Comments for Author box below.
Reviewer #1:

- Yes

Reviewer #2:

- No

Reproducibility
Were experiments performed using adequate number of biological replicates?
If 'No', please indicate reasons in Comments for Author box below.
Reviewer #1:

- Yes

© 2026. Published by The Company of Biologists under the terms of the Creative Commons Attribution License (https://creativecommons.org/licenses/by/4.0/).

Reviewer #2:
- No

Is there sufficient raw data to assess the rigor of the analysis?
If 'No', please indicate reasons in Comments for Author box below.
Reviewer #1:
- Yes

Reviewer #2:
- No

Does the methods section provide sufficient detail to permit reproducibility?
If 'No', please indicate reasons in Comments for Author box below.
Reviewer #1:
- Yes

Reviewer #2:
- No

Completeness
Are the manuscript's conclusions supported by the data?
If 'No', please indicate reasons in Comments for Author box below.
Reviewer #1:
- Yes

Reviewer #2:
- No

Scholarship
Do the authors cite and discuss the merits of data that would argue for and against their conclusion?
If 'No', please indicate reasons in Comments for Author box below.
Reviewer #1:
- Yes

Reviewer #2:
- Yes

Does the manuscript title & abstract accurately reflect the contents of the manuscript, without hyperbole?
If 'No', please indicate reasons in Comments for Author box below.
Reviewer #1:
- Yes

Reviewer #2:
- No

Resubmission

Author response to reviewers' comments

© 2026. Published by The Company of Biologists under the terms of the Creative Commons Attribution License (https://creativecommons.org/licenses/by/4.0/).

Comments from the Reviewers:

Reviewer 1: In this manuscript, Weaver et al. use a bilateral adrenalectomy/corticosterone-replacement design to ask whether glucocorticoids regulate pro-inflammatory cytokine gene expression (Il1b, Tnf) in brain and peripheral tissues of male mice exposed to acute sleep fragmentation. The principal findings are that pharmacologically elevated corticosterone broadly suppresses cytokine expression, that differences between sham and adrenalectomized animals are confined to a few tissues, and that sleep fragmentation itself produced essentially no inflammatory effect. The study is technically competent, the analyses are appropriate, and I particularly commend the authors for reporting the null sleep-fragmentation result openly. My substantive comments concern the framing of the work rather than its conduct, and I would support publication after these revisions.

Major
1. The manuscript is framed throughout as a test of glucocorticoid regulation of the inflammatory response to sleep fragmentation, but sleep fragmentation produced little or no such response in this cohort. The experiment therefore does not, in practice, address that question. It does, however, clearly demonstrate that elevated corticosterone suppresses cytokine gene expression across most tissues. I would encourage the authors to foreground this result and to revise the title and the concluding sentence of the Abstract so that the central claim concerns the anti-inflammatory action of corticosterone, with sleep fragmentation as the experimental context rather than the response being modulated. This requires no new data.

Thank you for this suggestion. We have now set-up the paper so that the unexpected result of there being no effect from sleep fragmentation is conveyed in the new title, intro, discussion, and abstract.

2. The replacement protocol produced circulating corticosterone markedly above the intact physiological range, so the strong suppressive effects are best described as pharmacological rather than as a physiological replacement. The authors note this in the Discussion; I ask that the Abstract and Conclusions reflect it consistently, and that the sham-versus-ADX contrast be presented explicitly as the study's window onto physiological glucocorticoid action, an effect detectable in only a small number of tissues.

Thank you. We have now altered the abstract and the conclusions to highlight that only a small number of tissues were affected by glucocorticoids at physiological levels.

3. All animals, including the sham group, underwent surgery with approximately one week of recovery. Residual surgical and HPA activation is a plausible explanation for the absent sleep-fragmentation effect, given that the authors have reported such effects previously. A brief acknowledgement of the lack of a non-surgical control would strengthen the Discussion.

Thank you. This is an excellent point and we have now acknowledged in the discussion that this potential confound could be addressed with the addition of an unmanipulated control.

4. The hypothalamic TNF-α interaction, in which corticosterone exerted opposing effects under sleep-fragmentation and control conditions, is the most novel observation in the study and currently receives limited treatment. I suggest a short paragraph on biphasic, state-dependent glucocorticoid action, and a clarification of the corresponding entry in Table 1, where the dual arrows are difficult to interpret as presented.

Thank you for this comment. We have now expanded the paragraph involving biphasic, state-dependent glucocorticoid actions, and have done a better job with explain the dual arrows in Table 1.

Minor
5. Two reported F-statistics have error degrees of freedom inconsistent with the other effects in the same ANOVA and appear to be typographical errors. Please reconcile.

Thank you. We have fixed these errors

© 2026. Published by The Company of Biologists under the terms of the Creative Commons Attribution License (https://creativecommons.org/licenses/by/4.0/).

6. Abstract: "glucocorticoids… has a suppressive effect" should read have.

Corrected. Thank you.
7. Typographical corrections: "respectfully" → respectively; "son serum" → on (Figure 2 legend); "supressed" → suppressed; and "stratum" → presumably striatum.

Corrected and many thanks.

Requires the narrative to be aligned with the results. With the framing adjustments above I would be happy to see it published.

Thank you for your assistance!

Reviewer 2: The experimental question is important and the adrenalectomy/replacement design is appropriate in principle for testing glucocorticoid involvement. However, this study suffers from several important limitations that preclude publication. First, it does not address the central question as it is currently framed. The major problem is that sleep fragmentation did not reliably induce the expected pro- inflammatory phenotype. This substantially weakens the claim that the study identifies glucocorticoid mediation of inflammatory responses to sleep fragmentation. A more conservative interpretation would be that pharmacological corticosterone replacement suppresses cytokine transcript expression in a tissue-dependent manner.’

Thank you for this observation. We agree that we cannot claim that glucocorticoids specifically mediate inflammation among SF mice, and have therefore altered conclusions drawn and the reworded the title and abstract.

There are several weaknesses in the design and reporting. First, only male mice were used, which limits inference of the study: sleep, HPA-axis biology and immune signalling, all show sex-dependent effects.

We agree that also studying females would benefit the study and have acknowledged this in the discussion.

The corticosterone replacement group appears to have reached pharmacological rather than physiological levels, making inference of normal glucocorticoid regulation impossible.

Even though the corticosterone replacement is pharmacological, the comparison between ADX and sham mice is within the physiological range of these mice.

Incidentally, the study would benefit from clearer evidence that the sleep-fragmentation protocol produced equivalent disruption across groups, especially given that adrenalectomy and corticosterone replacement could themselves alter sleep, arousal or stress responsiveness.

This is an excellent point. We have now acknowledged in the discussion that glucocorticoid treatment could affect how mice response to sleep fragmentation.

The statistical analysis is not fully convincing as reported. The study includes multiple tissues, two cytokine transcripts, corticosterone measurements and numerous post hoc contrasts, but there does not appear to be a clear correction or control strategy for multiple testing across the full family of outcomes.

We appreciate the reviewer's concern regarding the potential for inflated Type I error arising from multiple outcome measures. Our primary analyses were conducted using planned univariate models for each biological endpoint, with Tukey-adjusted post hoc comparisons applied to control for multiple pairwise testing within each analysis. We chose this approach because the individual outcomes (cytokine expression in distinct tissues and circulating corticosterone concentrations) represent biologically distinct endpoints rather than repeated measures of a single construct.

© 2026. Published by The Company of Biologists under the terms of the Creative Commons Attribution License (https://creativecommons.org/licenses/by/4.0/).

While multivariate approaches such as MANOVA can, in principle, account for correlations among outcomes and reduce the number of statistical tests, their utility depends on adequate sample sizes relative to the number of dependent variables. In vertebrate biomedical research, sample sizes are necessarily constrained by ethical considerations embodied in the principles of Replacement, Reduction, and Refinement (the 3Rs). As a result, studies of this type often lack sufficient statistical power to support robust multivariate analyses, particularly when multiple treatment groups and outcome variables are included. Under such circumstances, MANOVA can become unstable and may substantially reduce the ability to detect biologically meaningful effects.

We have therefore retained the univariate analytical framework, which is commonly employed in preclinical biomedical research, while applying appropriate corrections for multiple pairwise comparisons within each endpoint. We acknowledge that testing multiple biological outcomes increases the overall possibility of Type I error across the study and have added this consideration as a limitation in the Discussion. Importantly, the major conclusions are based on consistent patterns observed across related inflammatory markers and tissues rather than on isolated statistically significant findings.

The manuscript should also clarify whether cage or sleep-fragmentation chamber was treated as a potential experimental unit or blocking factor, since animals were housed in groups and the manipulation may not be fully independent at the individual-animal level.

We thank the reviewer for highlighting the importance of clarifying the experimental unit and potential clustering effects. We agree that housing conditions and sleep-fragmentation exposure could introduce non-independence if cage or chamber were acting as shared environmental units.

Unfortunately, individual cage assignments were not consistently recorded, which precludes post hoc evaluation of cage- or chamber-level clustering effects. We have now explicitly acknowledged this limitation in the manuscript. All animals were treated as independent experimental units in the primary analyses, and this assumption is now clearly stated in the Methods. We also note in the Discussion that the inability to account for potential cage or chamber effects may have contributed to variability in the data and should be considered when interpreting the findings. While this limitation is not uncommon in studies employing group-housed rodent designs, it is nonetheless important and has been transparently addressed.

Similarly, the biological replication is a concern. Final group sizes were small and uneven, particularly in the adrenalectomy groups, and no a priori power analysis or effect-size justification is provided. This is especially problematic for detecting sleep × corticosterone interactions. The absence of sleep-fragmentation effects may therefore reflect inadequate power, surgical recovery effects, or both. At the very least it would be expected that there was a power justification, clarity over exclusion criteria and timing, and ideally show individual-level data for all endpoints.

We appreciate the reviewer's concern regarding biological replication and statistical power. Sample sizes were determined based on prior studies employing similar adrenalectomy and sleep-fragmentation procedures and were constrained by the substantial mortality and attrition associated with adrenalectomy surgery. We agree that the final group sizes were smaller and somewhat unbalanced, particularly within the adrenalectomized groups, which may have reduced statistical power to detect modest main effects or interactions.

To improve transparency, we have revised the Methods section to provide information regarding animal exclusions, attrition, and the timing of these events. We have also clarified that animals were excluded only according to predefined criteria (e.g., surgical complications, failure to recover adequately, or technical issues affecting sample collection/analysis).

We acknowledge in the Discussion that the absence of significant sleep fragmentationeffects, particularly for sleep × corticosterone interactions, should be interpreted cautiously because the study may have been underpowered to detect small-to-moderate effects. Nevertheless,

© 2026. Published by The Company of Biologists under the terms of the Creative Commons Attribution License (https://creativecommons.org/licenses/by/4.0/).

several corticosterone-related effects were detected despite these sample sizes, suggesting that the experimental design retained sufficient sensitivity to identify biologically meaningful changes in multiple endpoints. We have therefore tempered our conclusions and explicitly noted that future studies with larger cohorts will be needed to more definitively evaluate potential interaction effects.

The methods have several important details missing, or a lesat, incompletely reported. The authors state that tissue samples were coded so that gene-expression analyses were blinded, but did they blind all of the allocation to experimental condition, surgery, sample collection, tissue dissection, exclusion decisions and statistical analysis? or how were these randomised?

No. The study only blinded the tissue samples for gene expression analysis. Mice were randomly assigned to experimental condition/surgery.

It should also report whether sample collection and processing order were counterbalanced across groups. This is critical detail because corticosterone and inflammatory transcripts are strongly time-sensitive, and although it states that collection was performed at 0800, a single sampling time and unclear collection order limit interpretation.

Yes. Sample collection and processing order were counter-balanced across groups.

The conclusions are much too broad. The data support tissue-dependent suppression of cytokine transcript expression by high corticosterone replacement more strongly than they support glucocorticoid mediation of sleep-fragmentation-induced inflammation.The title overstates the result, since the manuscript reports little evidence that sleep fragmentation itself increased pro-inflammatory responses. The abstract is ok, but the title should reflect the actual findings.

We have altered the title so that it more accurately reflects the results.

First decision letter

MS ID#: bio.062757

MS Title: Pharmacological corticosterone suppresses pro-inflammatory cytokine gene expression in a tissue-dependent manner independent of sleep fragmentation in male mice

Authors: Hunter Weaver, Kimberly A. Turner, Van Thuan Nguyen and Noah Ashley

I am happy to tell you that your manuscript has been accepted for publication in Biology Open, pending our standard publication integrity checks. It was accepted on 22nd June 2026.

Reviewer 1

Comments for the author

I still have some reservations about the design, but the authors acknowledge these as limitations in the discussion. The authors have addressed all of my additional comments and concerns, and the paper is now suitable for publication.

Reviewer's Responses to Questions

Experimental quality
Are experiments performed using appropriate methods that will answer the question (or test the hypothesis or support the observations) posed by the authors? Is the right tool used for the job?
If 'No', please indicate reasons in Comments for Author box below.
Reviewer #1:

© 2026. Published by The Company of Biologists under the terms of the Creative Commons Attribution License (https://creativecommons.org/licenses/by/4.0/).

- Yes

Reviewer #2:
- Yes

Were the data analyzed using appropriate statistical tests?
If 'No', please indicate reasons in Comments for Author box below.
Reviewer #1:
- Yes

Reviewer #2:
- Yes

Reproducibility
Were experiments performed using adequate number of biological replicates?
If 'No', please indicate reasons in Comments for Author box below.
Reviewer #1:
- Yes

Reviewer #2:
- Yes

Is there sufficient raw data to assess the rigor of the analysis?
If 'No', please indicate reasons in Comments for Author box below.
Reviewer #1:
- Yes

Reviewer #2:
- Yes

Does the methods section provide sufficient detail to permit reproducibility?
If 'No', please indicate reasons in Comments for Author box below.
Reviewer #1:
- Yes

Reviewer #2:
- Yes

Completeness
Are the manuscript's conclusions supported by the data?
If 'No', please indicate reasons in Comments for Author box below.
Reviewer #1:
- Yes

Reviewer #2:
- Yes

Scholarship
Do the authors cite and discuss the merits of data that would argue for and against their conclusion?
If 'No', please indicate reasons in Comments for Author box below.
Reviewer #1:
- Yes

© 2026. Published by The Company of Biologists under the terms of the Creative Commons Attribution License (https://creativecommons.org/licenses/by/4.0/).

Reviewer #2:

- Yes

Does the manuscript title & abstract accurately reflect the contents of the manuscript, without hyperbole?
If 'No', please indicate reasons in Comments for Author box below.
Reviewer #1:

- Yes

Reviewer #2:

- Yes

© 2026. Published by The Company of Biologists under the terms of the Creative Commons Attribution License (https://creativecommons.org/licenses/by/4.0/).
